# Supplementary material for: CRISPR prime editing for unconstrained correction of oncogenic KRAS variants
Source: Commun Biol. 2023 Jun 30;6:681. doi: 10.1038/s42003-023-05052-1 (PMC10313713; doi:10.1038/s42003-023-05052-1)
Supplement: Supplementary file 2 — Supplementary Information [file 42003_2023_5052_MOESM2_ESM.pdf]

Supplementary Information

CRISPR prime editing for unconstrained correction of oncogenic *KRAS* variants.

Gayoung Jang[1, 2], Jiyeon Kweon[1, 2], Yongsub Kim[1, 2, *]

[1] Department of Cell and Genetic Engineering, Asan Medical Institute of Convergence Science and Technology, Asan Medical Center, University of Ulsan College of Medicine, Seoul 05505, Republic of Korea
[2] Stem Cell Immunomodulation Research Center, University of Ulsan College of Medicine, Seoul 05505, Republic of Korea

* Corresponding author: Yongsub Kim, PhD (email: yongsub1.kim@gmail.com)

Table of contents

Supplementary Figure 1. *KRAS* correction frequency of *KRAS*-#1 universal pegRNA in HEK293T/17-*KRAS* library cells.
Supplementary Figure 2. Optimization of pegRNAs to generate *KRAS* heterozygous HEK293T/17 cells.
Supplementary Figure 3. *KRAS* correction frequencies in *KRAS* heterozygous HEK293T/17 cells.
Supplementary Figure 4. *KRAS* correction with pegRNA capable of inducing silent mutation in G12V heterozygous HEK293T/17 cells.
Supplementary Figure 5. Analysis of potential off-target effects of the universal epegRNA.
Supplementary Table 1. Substitution and indel frequencies at endogenous *KRAS* sites in HEK293T/17-*KRAS* library cells.
Supplementary Table 2. Prime editing and indel frequencies in HEK293T/17-*KRAS* heterogenous cells.
Supplementary Table 3. List of potential off-target sites.
Supplementary Table 4. *KRAS*-mutant target sequences used in HEK293T/17-*KRAS* library cells.
Supplementary Table 5. Sequences of gRNA and pegRNAs used in this study.
Supplementary Table 6. Primer sequences used in this study.

PBS (nt)	9	11	13	9	11	13	9	11	13
RTT (nt)	10			13			16		
G12V	0.2	0.0	0.1	0.0	0.7	0.7	0.8	0.0	0.5
G12D	0.5	0.5	0.4	2.3	0.9	0.8	1.2	0.7	0.7
G12A	0.0	0.2	0.1	1.2	0.3	1.2	0.0	0.6	0.7
G12C	0.1	0.7	0.4	1.5	1.9	1.5	0.8	0.7	0.6
G12R	0.2	0.9	0.6	1.3	1.3	2.9	0.8	0.8	1.0
G12S	0.6	1.1	0.9	2.1	1.5	0.5	0.0	0.9	0.7
G13C	n.a.	n.a.	n.a.	0.9	0.0	0.0	0.0	0.6	0.0
G13S	n.a.	n.a.	n.a.	0.0	0.7	0.0	0.0	0.4	0.0
G13R	n.a.	n.a.	n.a.	0.1	0.1	0.6	0.6	0.1	0.1
G13V	n.a.	n.a.	n.a.	0.0	0.0	0.2	0.0	0.1	0.1
G13D	n.a.	n.a.	n.a.	0.0	0.0	0.6	0.0	0.0	0.0
G13A	n.a.	n.a.	n.a.	0.3	0.2	0.4	0.0	0.0	0.1

Supplementary Figure 1. *KRAS* correction frequency of *KRAS*-#1 universal pegRNA in HEK293T/17-KRAS library cells.

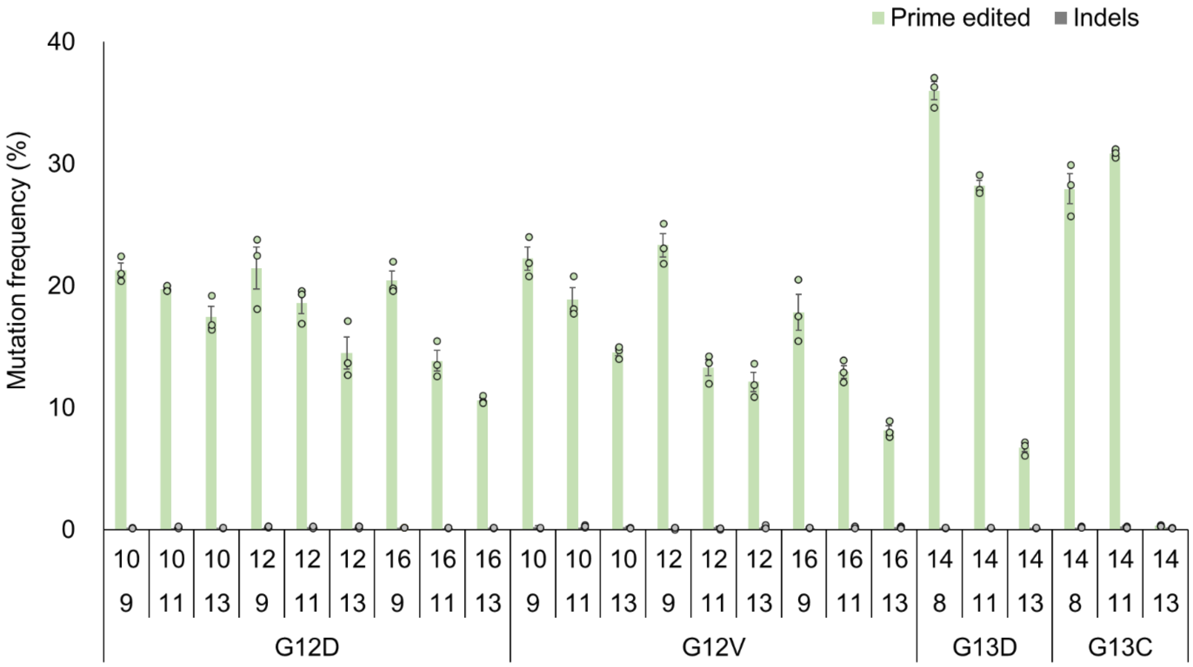

Supplementary Figure 2. Optimization of pegRNAs to generate *KRAS* heterozygous HEK293T/17 cells. Error bar means s.e.m. of *n=3* biological triplicate samples.

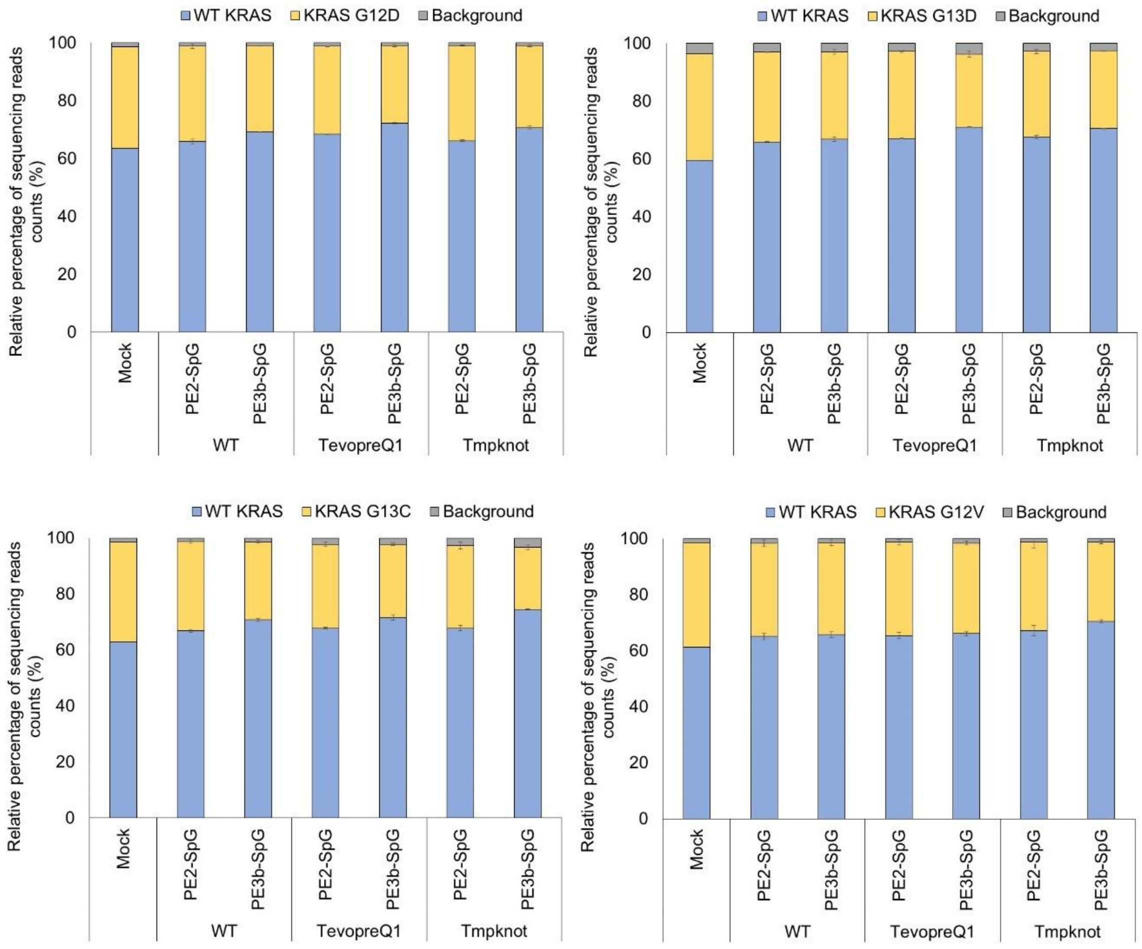

Supplementary Figure 3. *KRAS* correction frequencies in *KRAS* heterozygous HEK293T/17 cells. Target sites were amplified and analzed by targeted-deep sequencing. Relative counts of sequencing reads were calculated from total number of sequencing reads of wild-type *KRAS* sequences and mutant *KRAS* sequences. Error bar means s.e.m. of *n=3* biological triplicate samples.

KRAS G12V mutant alleles of HEK293T/17 heterogenous cells

```
   E  Y  K  L  V  V  V  G  A  V  G  V  G  K  S  A  L  T
5'-GAATATAAACTTGTGGTAGTTGGAGCTGTTGGCGTAGGCAAGAGTGCCTTGACGA-3'
3'-CTTATATTTGAACACCATCAACCTCGACAACCGCATCCGTTCTCACGGAACTGCT-5'
```

Prime editing using the universal pegRNA containing a silent mutation

```
   E  Y  K  L  V  V  V  G  A  G  G  V  G  K  S  A  L  T
5'-GAATATAAACTTGTGGTAGTTGGAGCAGGTGGCGTAGGCAAGAGTGCCTTGACGA-3'
3'-CTTATATTTGAACACCATCAACCTCGTCCACCGCATCCGTTCTCACGGAACTGCT-5'
```

Untreated	TAGTTGGAGCTGGTGGCGTAGGCAA	(2434/4029)	60.4%
	TAGTTGGAGCTGTTGGCGTAGGCAA	(1519/4029)	37.7%
	TAGTTGGAGCAGGTGGCGTAGGCAA	(0/4029)	0.0%
	TAGTTGGAGCAGTTGGCGTAGGCAA	(0/4029)	0.0%
pegRNA treated	TAGTTGGAGCTGGTGGCGTAGGCAA	(2889/4738)	61.0%
	TAGTTGGAGCTGTTGGCGTAGGCAA	(1561/4738)	32.9%
	TAGTTGGAGCAGGTGGCGTAGGCAA	(206/4738)	4.3%
	TAGTTGGAGCAGTTGGCGTAGGCAA	(0/4738)	0.0%

Supplementary Figure 4. *KRAS* correction with pegRNA capable of inducing silent mutation in G12V heterozygous HEK293T/17 cells. The pegRNA could induce T:A to A:T silent mutation at alanine 11 residue and correct *KRAS* G12V mutation. The silent mutation and *KRAS* G12V mutation were highlighted in blue and red, respectively, and the spacer sequences of pegRNA were boxed. NGS sequencing results showed that *KRAS* correction was accompanied by the silent mutation, indicating that *KRAS* correction was induced by prime editing.

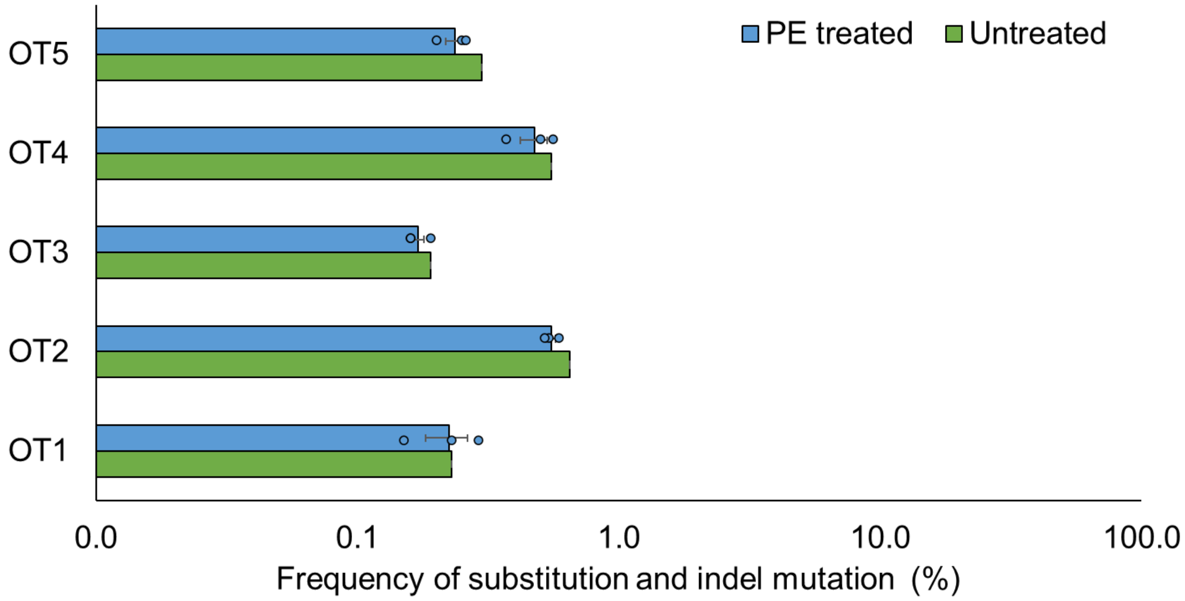

Supplemantary Figure 5. Analysis of potential off-target effects of the universal epegRNA. Five potential-offtarget sites were selected *in silico* and the universal epegRNA was transfected into HEK293T/17-*KRAS* G12V cells with PEmax-SpG-hMLH1dn. Targeted-deep sequencing results showed that there were no significant off-target mutations in the five potential off-target sites. The potential off-target sites were listed in Supplementary Table 3. Error bar means s.e.m. of biological triplicate.

Supplementary Table 1. Substitution and indel frequencies at endogenous *KRAS* sites in HEK293T/17-*KRAS* library cells.

			Replicate 1		Replicate 2	
**pegRNA names**	**PBS length (nt)**	**RTT length (nt)**	**Substitutions (%)**	**Indels (%)**	**Substitutions (%)**	**Indels (%)**
Universal pegRNA #2-1	9	10	1.3%	0.1%	1.3%	0.1%
Universal pegRNA #2-2	11	10	1.3%	0.1%	1.3%	0.1%
Universal pegRNA #2-3	13	10	1.5%	0.1%	1.2%	0.0%
Universal pegRNA #2-4	9	13	1.3%	0.0%	1.2%	0.0%
Universal pegRNA #2-5	11	13	1.5%	0.1%	1.4%	0.0%
Universal pegRNA #2-6	13	13	1.4%	0.0%	1.5%	0.0%
Universal pegRNA #2-7	9	16	1.5%	0.0%	1.2%	0.0%
Universal pegRNA #2-8	11	16	1.4%	0.1%	1.6%	0.0%
Universal pegRNA #2-9	13	16	1.4%	0.1%	1.4%	0.0%
Mock			1.6%	0.1%		

				Replicate 1		Replicate 2	
**PE systems**	**PBS length (nt)**	**RTT length (nt)**	**RNA scaffolds**	**Substitutions (%)**	**Indels (%)**	**Substitutions (%)**	**Indels (%)**
PE2-SpG	13	16	Wild-type	0.0%	0.0%	0.6%	0.0%
PE3b-SpG	13	16	Wild-type	0.8%	0.0%	0.6%	0.0%
PE2-SpG	13	16	TevopreQ1	0.8%	0.0%	0.5%	0.0%
PE3b-SpG	13	16	TevopreQ1	0.5%	0.0%	0.6%	0.1%
PE2-SpG	13	16	Tmpknot	0.6%	0.2%	0.6%	0.1%
PE3b-SpG	13	16	Tmpknot	0.5%	0.0%	0.6%	0.1%
Mock				0.8%	0.1%		

Supplementary Table 2. Prime editing and indel frequencies in HEK293T/17-*KRAS* heterogenous cells.

				G12D		G12V		G13D		G13C	
**PE systems**	**PBS length (nt)**	**RT length (nt)**	**RNA scaffolds**	**% of wild-type KRAS sequences**	**Indels (%)**	**% of wild-type KRAS sequences**	**Indels (%)**	**% of wild-type KRAS sequences**	**Indels (%)**	**% of wild-type KRAS sequences**	**Indels (%)**
PE2-SpG	13	16	Wild-type	66.6	0.0	65.1	0.0	66.1	0.0	66.1	0.0
PE3b-SpG	13	16	Wild-type	68.8	0.0	65.8	0.0	67.4	0.0	69.2	0.0
PE2-SpG	13	16	TevopreQ1	68.6	0.0	65.5	0.0	68.3	0.0	67.1	0.0
PE3b-SpG	13	16	TevopreQ1	71.0	0.0	66.2	0.0	71.2	0.0	70.1	0.0
PE2-SpG	13	16	Tmpknot	67.2	0.0	67.2	0.0	68.8	0.0	66.5	0.0
PE3b-SpG	13	16	Tmpknot	69.9	0.0	70.6	0.0	71.2	0.2	72.1	0.0
Mock				62		61.2		59.5		62.9	

Supplementary Table 3. List of potential off-target sites.

Target mutations		Target sequences (5' to 3')	PAM	Chr.	Mismatches (bp)
c.35T>G (G12V)	ON	CGTCAAGGCACTCTTGCCTA	CG	chr12	0
	OT1	CGTCActGCACTCTTGCCTA	GG	chr19	2
	OT2	aGaCAAGGCACTtTTGCCTA	TG	chr4	3
	OT3	CGTCAtGGgACTCTTtCCTA	CG	chr10	3
	OT4	aGTCAAGGCACTCcTaCCTA	TG	chr6	3
	OT5	CGTCActGCACTCTaGCCTA	GG	chr5	3

Supplementary Table 4. *KRAS*-mutant target sequences used in HEK293T/17-*KRAS* library cells.

KRAS **mutation**	***KRAS*** **mutant target sequence(5' to 3')**	**Barcode sequence**
G12V	ATGACTGAATATAAACTTGTGGTAGTTGGAGCTGtTGGCGTAGGCAAGAGTGCCTTGACG	CGAGTAAT
G12D	ATGACTGAATATAAACTTGTGGTAGTTGGAGCTGaTGGCGTAGGCAAGAGTGCCTTGACG	TCTCCGGA
G12A	ATGACTGAATATAAACTTGTGGTAGTTGGAGCTGcTGGCGTAGGCAAGAGTGCCTTGACG	AATGAGCG
G12C	ATGACTGAATATAAACTTGTGGTAGTTGGAGCTtGTGGCGTAGGCAAGAGTGCCTTGACG	GGAATCTC
G12R	ATGACTGAATATAAACTTGTGGTAGTTGGAGCTcGTGGCGTAGGCAAGAGTGCCTTGACG	TTCTGAAT
G12S	ATGACTGAATATAAACTTGTGGTAGTTGGAGCTaGTGGCGTAGGCAAGAGTGCCTTGACG	ACGAATTC
G13C	ATGACTGAATATAAACTTGTGGTAGTTGGAGCTGGTtGCGTAGGCAAGAGTGCCTTGACG	AGCTTCAG
G13S	ATGACTGAATATAAACTTGTGGTAGTTGGAGCTGGTaGCGTAGGCAAGAGTGCCTTGACG	GCGCATTA
G13R	ATGACTGAATATAAACTTGTGGTAGTTGGAGCTGGTcGCGTAGGCAAGAGTGCCTTGACG	CATAGCCG
G13V	ATGACTGAATATAAACTTGTGGTAGTTGGAGCTGGTGtCGTAGGCAAGAGTGCCTTGACG	TTCGCGGA
G13D	ATGACTGAATATAAACTTGTGGTAGTTGGAGCTGGTGaCGTAGGCAAGAGTGCCTTGACG	GCGCGAGA
G13A	ATGACTGAATATAAACTTGTGGTAGTTGGAGCTGGTGcCGTAGGCAAGAGTGCCTTGACG	CTATCGCT

Supplementary Table 5. Sequences of gRNA for PE3b, pegRNAs and epegRNAs used in this study.

gRNA or pegRNA name	Spacer sequence (5' to 3')	PAM	3' extension sequence (5' to 3')	PBS length (nt)	RTT length (nt)
G12D mutant pegRNA #1	AAACTTGTGGTAGTTGGAGC	TGG	GCCAtCAGCTCCAACTACC	9	10
G12D mutant pegRNA #2	AAACTTGTGGTAGTTGGAGC	TGG	GCCAtCAGCTCCAACTACCAC	11	10
G12D mutant pegRNA #3	AAACTTGTGGTAGTTGGAGC	TGG	GCCAtCAGCTCCAACTACCACAA	13	10
G12D mutant pegRNA #4	AAACTTGTGGTAGTTGGAGC	TGG	ACGCCAtCAGCTCCAACTACC	9	12
G12D mutant pegRNA #5	AAACTTGTGGTAGTTGGAGC	TGG	ACGCCAtCAGCTCCAACTACCAC	11	12
G12D mutant pegRNA #6	AAACTTGTGGTAGTTGGAGC	TGG	ACGCCAtCAGCTCCAACTACCACAA	13	12
G12D mutant pegRNA #7	AAACTTGTGGTAGTTGGAGC	TGG	GCCTACGCCAtCAGCTCCAACTACC	9	16
G12D mutant pegRNA #8	AAACTTGTGGTAGTTGGAGC	TGG	GCCTACGCCAtCAGCTCCAACTACCAC	11	16
G12D mutant pegRNA #9	AAACTTGTGGTAGTTGGAGC	TGG	GCCTACGCCATCAGCTCCAACTACCACAA	13	16
G12V mutant pegRNA #1	AAACTTGTGGTAGTTGGAGC	TGG	GCCAaCAGCTCCAACTACC	9	10
G12V mutant pegRNA #2	AAACTTGTGGTAGTTGGAGC	TGG	GCCAaCAGCTCCAACTACCAC	11	10
G12V mutant pegRNA #3	AAACTTGTGGTAGTTGGAGC	TGG	GCCAaCAGCTCCAACTACCACAA	13	10
G12V mutant pegRNA #4	AAACTTGTGGTAGTTGGAGC	TGG	ACGCCAaCAGCTCCAACTACC	9	12
G12V mutant pegRNA #5	AAACTTGTGGTAGTTGGAGC	TGG	ACGCCAaCAGCTCCAACTACCAC	11	12
G12V mutant pegRNA #6	AAACTTGTGGTAGTTGGAGC	TGG	ACGCCAaCAGCTCCAACTACCACAA	13	12
G12V mutant pegRNA #7	AAACTTGTGGTAGTTGGAGC	TGG	GCCTACGCCAaCAGCTCCAACTACC	9	16
G12V mutant pegRNA #8	AAACTTGTGGTAGTTGGAGC	TGG	GCCTACGCCAaCAGCTCCAACTACCAC	11	16
G12V mutant pegRNA #9	AAACTTGTGGTAGTTGGAGC	TGG	GCCTACGCCAaCAGCTCCAACTACCACAA	13	16
G13C mutant pegRNA #1	CTTGTGGTAGTTGGAGCTGG	TGG	TGCCTACGCaACCAGCTCCAAC	8	14
G13C mutant pegRNA #2	CTTGTGGTAGTTGGAGCTGG	TGG	TGCCTACGCaACCAGCTCCAACTAC	11	14
G13C mutant pegRNA #3	CTTGTGGTAGTTGGAGCTGG	TGG	TGCCTACGCaACCAGCTCCAACTACCA	13	14
G13D mutant pegRNA #1	CTTGTGGTAGTTGGAGCTGG	TGG	TGCCTACGtCACCAGCTCCAAC	8	14
G13D mutant pegRNA #2	CTTGTGGTAGTTGGAGCTGG	TGG	TGCCTACGtCACCAGCTCCAACTAC	11	14
G13D mutant pegRNA #3	CTTGTGGTAGTTGGAGCTGG	TGG	TGCCTACGtCACCAGCTCCAACTACCA	13	14
Universal pegRNA #1-1	TATAAACTTGTGGTAGTTGG	AG	AccAGCTCCAACTACCACA	9	10
Universal pegRNA #1-2	TATAAACTTGTGGTAGTTGG	AG	AccAGCTCCAACTACCACAAG	11	10
Universal pegRNA #1-3	TATAAACTTGTGGTAGTTGG	AG	AccAGCTCCAACTACCACAAGTT	13	10
Universal pegRNA #1-4	TATAAACTTGTGGTAGTTGG	AG	GccAccAGCTCCAACTACCACA	9	13
Universal pegRNA #1-5	TATAAACTTGTGGTAGTTGG	AG	GccAccAGCTCCAACTACCACAAG	11	13
Universal pegRNA #1-6	TATAAACTTGTGGTAGTTGG	AG	GccAccAGCTCCAACTACCACAAGTT	13	13
Universal pegRNA #1-7	TATAAACTTGTGGTAGTTGG	AG	TACGccAccAGCTCCAACTACCACA	9	16
Universal pegRNA #1-8	TATAAACTTGTGGTAGTTGG	AG	TACGccAccAGCTCCAACTACCACAAG	11	16
Universal pegRNA #1-9	TATAAACTTGTGGTAGTTGG	AG	TACGccAccAGCTCCAACTACCACAAGTT	13	16
Universal pegRNA #2-1	CGTCAAGGCACTCTTGCCTA	CG	ggTggCGTAGGCAAGAGTG	9	10
Universal pegRNA #2-2	CGTCAAGGCACTCTTGCCTA	CG	ggTggCGTAGGCAAGAGTGCC	11	10
Universal pegRNA #2-3	CGTCAAGGCACTCTTGCCTA	CG	ggTggCGTAGGCAAGAGTGCCTT	13	10
Universal pegRNA #2-4	CGTCAAGGCACTCTTGCCTA	CG	GCTggTggCGTAGGCAAGAGTG	9	13
Universal pegRNA #2-5	CGTCAAGGCACTCTTGCCTA	CG	GCTggTggCGTAGGCAAGAGTGCC	11	13
Universal pegRNA #2-6	CGTCAAGGCACTCTTGCCTA	CG	GCTggTggCGTAGGCAAGAGTGCCTT	13	13
Universal pegRNA #2-7	CGTCAAGGCACTCTTGCCTA	CG	GGAGCTggTggCGTAGGCAAGAGTG	9	16
Universal pegRNA #2-8	CGTCAAGGCACTCTTGCCTA	CG	GGAGCTggTggCGTAGGCAAGAGTGCC	11	16
Universal pegRNA #2-9	CGTCAAGGCACTCTTGCCTA	CG	GGAGCTggTggCGTAGGCAAGAGTGCCTT	13	16
PE3b Correction gRNA #1	GCACTCTTGCCTACGCCACC	AG	N/A	N/A	N/A
PE3b Correction gRNA #2	GTGGTAGTTGGAGCTGGTGG	CG	N/A	N/A	N/A
pegRNA-silent mutation	CGTCAAGGCACTCTTGCCTA	CG	GGAGCaggTggCGTAGGCAAGAGTGCCTT	13	16

epegRNA name	Spacer sequence (5' to 3')	PAM	3' extension sequence (5' to 3')	PBS length (nt)	RTT length (nt)	Linker
TevopreQ1 epegRNA #2-9	CGTCAAGGCACTCTTGCCTA	CG	GGAGCTggTggCGTAGGCAAGAGTGCCTT	13	16	CTTAAGTA
Tmpknot epegRNA #2-9	CGTCAAGGCACTCTTGCCTA	CG	GGAGCTggTggCGTAGGCAAGAGTGCCTT	13	16	TAATTATA

Supplementary Table 6. Primer sequences used in this study.

Target sites	Primer name	Forward primers	Reverse primers
Endogenous *KRAS*	1st	CTTAAGCGTCGATGGAGGAG	CCCTGACATACTCCCAAGGA
	2nd	ACACTCTTTCCCTACACGACGCTCTTCCGATCTAGGCCTGCTGAAAATGACTG	GTGACTGGAGTTCAGACGTGTGCTCTTCCGATCTTCATGAAAATGGTCAGAGAAACC
Lenti_*KRAS* sequence (HEK293T/17-*KRAS* library cells)	1st	GTGTACGGTGGGAGGCCTAT	GTCCGTCTGCGAGGGTACTA
	2nd	ACACTCTTTCCCTACACGACGCTCTTCCGATCTGCTCGTTTAGTGAACCGTCAG	GTGACTGGAGTTCAGACGTGTGCTCTTCCGATCTACGTGAAGAATGTGCGAGA
Universal pegRNA #2 OT1	2nd	ACACTCTTTCCCTACACGACGCTCTTCCGATCTTGTGCCAGTAGTCCCAGCTA	GTGACTGGAGTTCAGACGTGTGCTCTTCCGATCTTCTACAAAAGAACCCATGGAAGG
Universal pegRNA #2 OT2	2nd	ACACTCTTTCCCTACACGACGCTCTTCCGATCTTGGATTTTGGACTTGCATGA	GTGACTGGAGTTCAGACGTGTGCTCTTCCGATCTGTCCCCCAAAGCCTTAACTC
Universal pegRNA #2 OT3	2nd	ACACTCTTTCCCTACACGACGCTCTTCCGATCTTGAGAATGTCATCGGCACAC	GTGACTGGAGTTCAGACGTGTGCTCTTCCGATCTTTCAGCGCTTTCAGTGTTCA
Universal pegRNA #2 OT4	2nd	ACACTCTTTCCCTACACGACGCTCTTCCGATCTTCACACCCTTCAAATTGGCA	GTGACTGGAGTTCAGACGTGTGCTCTTCCGATCTTTGCCTGCTGATGGACATTA
Universal pegRNA #2 OT5	2nd	ACACTCTTTCCCTACACGACGCTCTTCCGATCTGCAGATACCCAGGAGAGCTT	GTGACTGGAGTTCAGACGTGTGCTCTTCCGATCTAGGATCCCTTGAGCGCTG
