# Supplementary material for: CRISPR prime editing for unconstrained correction of oncogenic KRAS variants
Source: Commun Biol. 2023 Jun 30;6:681. doi: 10.1038/s42003-023-05052-1 (PMC10313713; doi:10.1038/s42003-023-05052-1)
Supplement: Supplementary file 3 — Description of Additional Supplementary Files [file 42003_2023_5052_MOESM3_ESM.pdf]

Description of Additional Supplementary Files

File name: Supplementary Data 1
Description: Source data for the plots and graphs in the figures.
